# Timing of percutaneous vertebroplasty in the management of osteoporotic vertebral compression fractures: a retrospective cohort study

**DOI:** 10.3389/fsurg.2025.1539057

**Published:** 2025-05-14

**Authors:** Huawei Wei, Qiuxue Zhang, Bing Song, Hongwei Yue, Jing Li, Hongjian Zhang, Yanming Wang

**Affiliations:** ^1^ Department of Orthopedics, Qilu Hospital Dezhou Hospital, Shandong University, Dezhou, Shandong, China; ^2^ Minimally Invasive Surgery Centre, Qilu Hospital Dezhou Hospital, Shandong University, Dezhou, Shandong, China

**Keywords:** osteoporotic vertebral compression fractures, percutaneous vertebroplasty, surgical timing, complications, patient outcomes

## Abstract

**Introduction:**

Osteoporotic vertebral compression fractures (OVCFs) are common in older populations, and cause pain and kyphosis, impacting patient quality of life. This study aimed to determine the optimal timing of percutaneous vertebroplasty (PVP) for the treatment of OVCFs.

**Methods:**

This retrospective cohort study included 120 older patients with OVCFs admitted to our hospital between January 2020 and December 2022. Patients were divided into three groups according to the timing of PVP surgery: Group A, within 7 days of the OVCF; Group B, 8–14 days after the OVCF; and Group C, 15–28 days after the OVCF. Preoperative and postoperative visual analog scale scores, the Oswestry Disability Index, vertebral height, Cobb angle, and complications were compared among the three groups.

**Results:**

PVP surgery within 7 days of the OVCF led to rapid pain relief and restoration of vertebral function; preoperative visual analog scale scores and the Oswestry Disability Index were significantly higher in this group compared with the other two groups, but no significant differences among the three groups were observed postoperatively. However, early surgery was also associated with a higher rate of bone cement leakage compared with the other two groups, whereas delayed PVP was associated with a greater incidence of deep vein thrombosis and urinary infections.

**Discussion:**

The timing of PVP surgery in the management of OVCFs impacts patient outcomes. Early surgical treatment may result in greater pain relief, improved vertebral function, and fewer complications, but patient-specific factors should be considered when determining the optimal surgical timing.

## Introduction

Osteoporosis is common in older individuals, and the increased bone fragility caused by this condition can result in osteoporotic fractures, with osteoporotic vertebral compression fractures (OVCFs) the most prevalent type. The incidence of new vertebral fractures is increasing annually owing to the aging global population, with approximately 1.4 million new cases of OVCF per year worldwide (1). OVCFs typically affect the thoracic and lumbar vertebrae, and are defined as fractures resulting from osteoporosis, in which the vertebral body loses height, leading to acute or chronic back pain and kyphosis, significantly impairing the quality of life of affected individuals. Treatment for OVCFs can be either conservative or surgical. Patients with mild symptoms or those unable to undergo surgery typically receive conservative treatment, such as bed rest, analgesia, back braces, and physiotherapy (2). However, these treatment methods are slow to take effect and of long duration, and are frequently associated with severe complications such as pendant pneumonia, bedsores, and deep vein thrombosis (2). Surgical options in the form of vertebral augmentation, namely percutaneous vertebroplasty (PVP) and percutaneous kyphoplasty, are widely used to treat OVCFs as they produce immediate pain relief. Percutaneous kyphoplasty is based on PVP with the addition of vertebrae expansion using a balloon to reduce the compression fracture and create a cavity for cement injection. As a result it is more effective than PVP in restoring vertebral body height and correcting kyphosis (3, 4). However, PVP is more widely used owing to its simplicity and low cost, and numerous studies have shown that treatment of OVCFs with PVP results in favorable clinical outcomes (5–9). Nevertheless, the optimal timing for surgical intervention remains to be established. Some patients choose to undergo surgery immediately after a fracture, whereas others prefer to try conservative treatment first and undergo surgery only if it is unsuccessful. Clinically, fractures are less stable immediately after they occur due to vertebral trabecular breakage and microfractures, which can cause acute pain. Early surgery can therefore significantly reduce pain, prevent further exacerbation of the fracture, and reduce the incidence of complications associated with prolonged periods in bed; however, the risk of cement leakage may be increased (10, 11). Late surgery may reduce this risk, but have less of an impact on pain and vertebrae height restoration (10, 11).

Patients with OVCFs often present with multiple comorbidities and are typically older. These factors render the timing of surgery critical for patient outcomes. Therefore, this study aimed to compare surgical outcomes and complications of patients with OVCFs according to the surgical timing. The study sought to test the following hypotheses: (1) all three surgical timings can achieve good outcomes and significantly alleviate patient pain, (2) earlier surgical intervention is associated with a more effective restoration of injured vertebral height, and (3) delayed surgery may increase the risk of complications.

## Method

### Study design

This retrospective study included 120 older patients with OVCFs who were treated at our hospital between January 2020 and December 2022. The Ethics Committee of the Qilu Hospital Dezhou Hospital, Shandong University approved the study protocol.The inclusion criteria were as follows: aged ≥55 years, significant lumbar pain, a visual analog scale (VAS) score ≥5 at admission, decreased bone mineral density (T score <−1), the presence of bone marrow edema in the diseased vertebrae on magnetic resonance imaging T2-weighted short tau inversion recovery sequences, no more than 28 days between the onset of new back pain and PVP, and a single level compression fracture of T5 or a lower lumbar vertebra as determined by MRI with no evidence of fracture mass in the spinal canal. The exclusion criteria were as follows: insignificant chest and waist pain or a VAS score <5, radiographic findings of a multilevel vertebral compression fracture, suspected underlying malignancy, spinal infection, spinal cord and nerve root injuries, destruction of the lumbar vertebra posterior wall with fracture mass present in the spinal canal, uncontrollable bleeding disorders, an infection near the fractured vertebrae, and an inability to undergo surgery due to severe physical diseases such as respiratory diseases (12). The patients were divided into three groups based on the number of days from the onset of pain due to the fracture, rather than the patient's choice of surgical timing: Group A: surgery performed within 7 days of the fracture onset, Group B: surgery performed 8–14 days after the fracture, Group C: surgery performed 15–28 days after the fracture. No patients showed radiographic signs of bone healing, such as callus formation, cortical thickening, trabecular bridging, or increased vertebral density, on preoperative imaging, indicating that the fractures remained in the acute or subacute stage. Additionally, all patients had persistent unrelieved low back pain. All patients in the study underwent PVP.

### Surgical procedure

All patients underwent preoperative examinations to exclude any contraindications for surgery; consultations with relevant departments were conducted if necessary. The surgical procedure was performed with the patient in the prone position, with the chest and buttocks elevated. Prior to surgery, the surgical area was disinfected, and the puncture entry point was determined under C-arm x-ray fluoroscopy. After the induction of local anesthesia, the puncture needle was inserted into the vertebral arch from the puncture site at an inward angle of approximately 30° to 45°, 1–3 mm outside the projection of the vertebral arch. The entry angle was adjusted according to the angle of the vertebral arch, and the needle was inserted until the tip of the needle exceeded 1/3 of the anterior middle of the diseased vertebra. The needle core was then removed, and a working channel was established.

**Figure 1:**
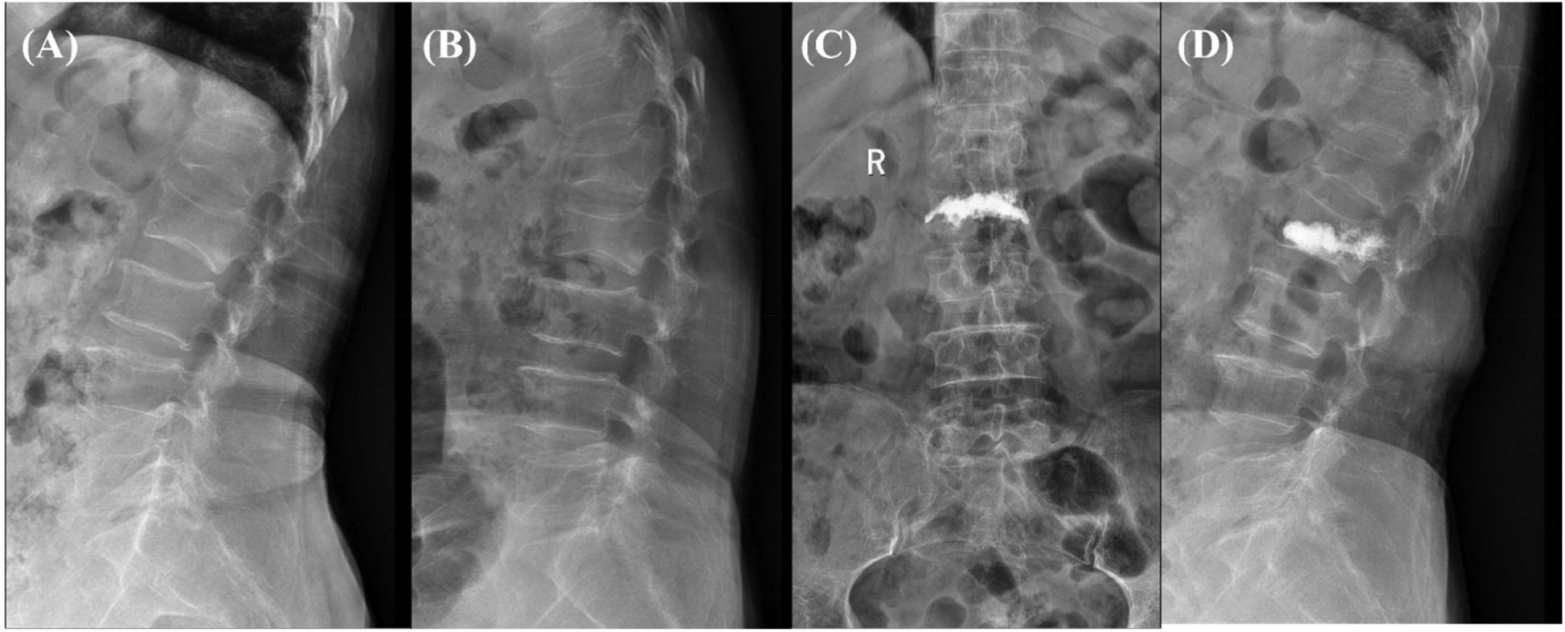
**(A,B)** preoperative x-ray showing an L2 vertebral compression fracture, characterized by significant vertebral body collapse and height loss. **(C,D)** Postoperative x-ray following vertebroplasty, demonstrating successful cement injection and filling of the fractured vertebra, with restored vertebral body height and complete cement filling. No complications, such as cement leakage, are observed. The patient showed good functional recovery post-surgery.

Equal proportions of polymethylmethacrylate bone cement powder and liquid were rapidly mixed and then injected under x-ray fluoroscopy. All procedures were performed by a single senior attending spine surgeon with extensive experience in percutaneous vertebroplasty (PVP), ensuring procedural consistency. The surgeon had undergone specialized training and had performed more than 100 similar cases. The injection was stopped when the vertebral body was full or the bone cement was diffused to approximately 4 mm anterior to the posterior wall of the vertebral body. Patients were closely monitored during bone cement injection, and the working channel was removed after bone cement solidification. Postoperative assessment of cement injection success was conducted using x-ray and CT scans to confirm uniform cement distribution within the fractured vertebra and to detect any leakage. A successful injection was defined as adequate cement filling without significant extravasation. In our study, all injections were deemed successful based on these imaging criteria, with no cases requiring additional intervention (Figure 1).

Immediately following surgery, all patients lay flat. They then spent 7–10 days in the hospital before discharge, during which time they were closely monitored for any complications and began rehabilitation. Following discharge, patients spent three months able to move around in bed while wearing a brace. All patients received standard anti-osteoporosis treatment consisting of calcium and vitamin D supplementation, as well as bisphosphonates (e.g., alendronate) during hospitalization and after discharge. This treatment was prescribed regardless of the presence of insufficiency fractures, as part of a comprehensive approach to fracture prevention in patients with osteoporosis.

### Clinical evaluation

Preoperative and postoperative VAS scores, the Oswestry Disability Index (ODI), the height of the anterior margin of the diseased vertebrae, and the Cobb angle between the superior endplate of the upper vertebra and the inferior endplate of the lower vertebra were compared among the three groups. The Cobb angle was measured using standard radiographs, with the angle formed between the line drawn along the superior endplate of the vertebra above the fractured vertebra and the line along the inferior endplate of the vertebra below the fracture site. This method allows for the assessment of the degree of kyphosis localized at the site of the osteoporotic vertebral compression fracture (OVCF). Postoperative complications were also recorded.

### Statistical analysis

Statistical analysis was performed using SPSS software version 25.0 (IBM Corporation, Armonk, NY, USA). Measurement data are presented as mean ± standard deviation, whereas count data are presented as number and proportion. The normality of the distribution of all measurement data was assessed using the Shapiro–Wilk test. Normally distributed data were compared using paired *t*-tests or one-way analysis of variance; non-normally distributed data were compared using non-parametric tests. Descriptive data and the incidence of complications were compared using the chi-square test. *P* < 0.05 was considered to indicate statistical significance.

## Results

### Patient characteristics

A total of 120 patients, 40 in each group, underwent PVP. Preoperative evaluations demonstrated no noteworthy contraindications for surgery in any of the groups. No significant differences among the groups were observed in terms of factors such as age, sex, body mass index, injured segment, and OVCF Genant classification (*P* > 0.05). Patient characteristics are presented in Table 1.

**Table 1:** Comparison of the general data.

Groups	Cases	Age	BMI	Sex		Genant grade		Cause of injury			Vertebral segment				
				Male	Female	Ⅱ	Ⅲ	traffic accident	fall	no obvious inducement	T12	L1	L2	L3	L4
Group A	40	68.69 ± 5.85	21.05 ± 3.52	16	24	19	21	5	12	23	13	14	6	4	3
Group B	40	66.93 ± 7.51	21.39 ± 2.83	18	22	17	23	3	15	22	15	12	5	5	3
Group C	40	67.25 ± 5.13	22.04 ± 2.82	16	24	18	22	4	15	21	16	12	6	4	2
F/*χ*^2^ *value	-	1.073	2.325	0.274*		0.202*		1.019*			1.298*				
*P* value	-	0.345	0.102	0.872		0.904		0.907			0.752				

### VAS scores and the ODI

Significant differences were observed in the preoperative VAS scores among the three groups (*P* < 0.05), with the highest preoperative VAS score in Group A (6.23 ± 1.06), followed by Group B (5.50 ± 1.10) and Group C (5.12 ± 1.02). Notably, the VAS scores in all three groups were substantially reduced at one day and three months postoperatively compared with preoperative levels, with no significant differences observed between the groups postoperatively (*P* > 0.05). The preoperative ODI were 36.10 ± 3.93 for Group A, 34.55 ± 4.35 for Group B, and 29.75 ± 4.72 for Group C (*P* < 0.05). The ODI of all three groups was notably reduced at one day and three months postoperatively compared with preoperative levels, with no significant differences observed between the groups postoperatively (*P* > 0.05). VAS and ODI data are shown in Table 2.

**Table 2:** Comparison of VAS scores and ODI scores.

Group	VAS score			ODI score		
	Preoperative	1 day	3 months	Preoperative	1 day	3 months
Group A	6.23 ± 1.06	2.19 ± 0.60	1.33 ± 0.51	36.10 ± 3.93	18.15 ± 2.29	13.58 ± 2.45
Group B	5.50 ± 1.10	2.07 ± 0.57	1.41 ± 0.54	34.55 ± 4.35	18.38 ± 2.43	13.90 ± 2.09
Group C	5.12 ± 1.02	2.25 ± 0.58	1.42 ± 0.47	29.75 ± 4.72	18.45 ± 2.26	13.13 ± 2.34
F value	6.028	0.970	0.361	7.22	0.180	1.147
*P* value	0.015	0.382	0.697	0.011	0.836	0.321

### Cobb angle and anterior height of diseased vertebrae

Preoperative Cobb angles were smallest in Group A (26.25° ± 5.21°), followed by Group B (27.98° ± 6.16°) and Group C (29.95° ± 5.60°) (*P* < 0.05). The Cobb angle was lower in all three groups at one day and three months postoperatively compared to preoperatively; however, there remained significant differences between the groups, with the smallest in Group A and largest in Group C (*P* < 0.05). Preoperative anterior vertebral body height was largest in Group A (28.98 ± 5.71 mm), followed by Group B (27.55 ± 5.87 mm) and Group C (26.15 ± 5.55 mm) (*P* < 0.05). The height of the anterior vertebral body was significantly increased in all three groups at one day and three months postoperatively, compared to preoperatively; however, there remained significant differences between the groups, with the largest value in Group A and the smallest in Group C (*P* < 0.05). Cobb angle and anterior height data are presented in Table 3.

**Table 3:** Comparison of cobb angle and anterior vertebral body margin height.

Group	Cobb angle (°)			Anterior vertebral body margin height (mm)		
	Preoperative	1 day	3 months	Preoperative	1 day	3 months
Group A	26.25 ± 5.21	10.07 ± 3.91	13.08 ± 4.08	28.98 ± 5.71	42.18 ± 4.58	38.22 ± 4.86
Group B	27.98 ± 6.16	12.88 ± 4.43	14.80 ± 4.94	27.55 ± 5.87	41.28 ± 5.76	37.18 ± 5.68
Group C	29.95 ± 5.60	16.88 ± 4.85	18.43 ± 4.33	26.15 ± 5.55	38.60 ± 5.07	35.60 ± 5.28
F value	4.264	2.970	2.361	3.446	5.198	2.800
*P* value	0.016	0.032	0.037	0.031	0.007	0.046

### Complications

In Group A, 10 instances of bone cement leakage were observed, compared with 5 in Group B and 3 in Group C (*P* < 0.05). Group A had 1 case of deep vein thrombosis and 2 cases of urinary infection, whereas Group C experienced 5 cases of deep vein thrombosis and 2 cases of urinary infection (*P* < 0.05). No significant differences were observed among the groups in terms of the number of adjacent vertebral fractures: 8 in Groups A and C and 7 in Group B (*P* > 0.05). Complications are listed in Table 4.

**Table 4:** Details of complications.

Group	Bone cement leakage (*n* = 40)	DVT (*n* = 40)	Urinary infection (*n* = 40)	Adjacent vertebral fractures (*n* = 40)
Group A	10/30	1/39	2/38	8/23
Group B	5/35	3/37	4/36	7/22
Group C	3/37	5/35	7/33	8/21
*χ*^2^value	9.028	6.839	7.229	2.050
*P* value	0.027	0.038	0.031	0.658

## Discussion

This study investigated the optimal timing of PVP for the treatment of OVCFs. The findings of the study revealed that early surgery (within seven days) facilitated rapid pain relief and restoration of vertebral function. Although the surgical timing did not significantly influence postoperative VAS scores and the ODI, early intervention shortened the duration of pain and decreased the incidence of complications associated with prolonged periods in bed. Injecting bone cement into the compressed vertebral body rapidly restores the stress structure and stability of the affected vertebrae, providing effective pain relief. This is due in part to the large amount of heat generated during the polymerization of the bone cement monomer, which causes necrosis of the peripheral nerve endings (4). Immediately after a fracture, the strength of the affected vertebra is reduced, bone trabeculae may become fragmented, and microfractures can occur, potentially leading to vertebral collapse or the accumulation of ‘silent’ microfractures. These changes can damage the lumbar dorsal fascia and increase the pain sensitivity of peripheral nerves (13–15). Surgical treatment performed during this period can therefore effectively alleviate pain.

After the immediate post-fracture period, the persistence of kyphosis and the accumulation of microfractures in the fractured vertebra may contribute to ongoing inflammation and increased bone resorption at the fracture site which can be definitively assessed through histopathological examination, imaging techniques such as MRI (especially T2-weighted sequences) and bone scintigraphy (16). Patients gradually adapt to this state of instability by significantly increasing their pain threshold. In this study, patients in Group A (operated on within 7 days of the fracture) experienced significantly lower VAS scores and better pain control compared to those in Group B (operated on 8–14 days after the fracture) and Group C (operated on 15–28 days after the fracture). Although patients in Group A had a significantly higher preoperative ODI than that of patients in the other two groups, this significant difference disappeared following surgery. These results suggest that early surgical treatment can relieve pain more quickly, improve lumbar spine function, and enhance quality of life. These findings are consistent with those of Ehsanian et al., who compared the pain outcomes of 126 patients who underwent PVP <6 weeks, 6–12 weeks, and >12 weeks after vertebral compression fractures and revealed that early intervention (<12 weeks) was associated with improved pain scores when compared to later intervention (>12 weeks); very early intervention (<6 weeks) conferred a greater advantage. In contrast to the study by Ehsanian et al., the present study limited the included patients to those operated on within 28 days of OVCF and compared the ODI and complications in the outcome measures. This provides more specific information about the relationship between surgical timing and patient outcomes, to assist clinicians in making better-informed decisions about the optimal timing of PVP, ultimately leading to improved patient outcomes and fewer complications (17).

The present study also demonstrated that the timing of surgery has an impact on the recovery and stability of the vertebral structure. The postoperative vertebral body anterior height and Cobb's angle achieved in Groups A and B were significantly better than those in Group C. Early intervention, within 7–14 days of the fracture, allows for more effective stabilization and kyphosis correction before significant bone healing or remodeling occurs. In contrast, delayed surgery (15–28 days post-fracture) may involve the formation of soft callus or trabecular healing, making it harder to restore vertebral height and alignment as the fracture site stabilizes. Early surgery reduces the risk of complications, such as vertebral collapse, worsening kyphosis, and muscle contractures, which can complicate correction and lead to worse outcomes. In addition, the timing of surgery played a crucial role in the occurrence of postoperative complications. Early surgical treatment was associated with a relatively high rate of cement leakage. The formation and further expansion of a fracture line within the vertebral body during PVP increases the likelihood of vertebral fracture, and so cement leakage (18–22). Over time, the fractured end of the compressed fracture block fills with new granulation tissue, reducing cement leakage (19–22). However, the delay before surgery also increases the incidence of deep vein thrombosis and urinary tract infection (23), owing to the prolonged period of preoperative bed rest. Therefore, altering the surgical timing changes not only the complication rate but the type of complications experienced. This should be carefully considered when deciding when to operate.

This study has certain limitations that should be acknowledged. First, the small sample size may affect the reliability of the results. Future analyses involving larger cohorts are necessary to improve the accuracy of the findings. Second, this study did not evaluate intraoperative factors, such as surgeon experience, surgical technique, and type of bone cement, which may also influence outcomes.

In conclusion, the timing of PVP surgery is crucial for the management of OVCFs. The findings of this study suggest that early surgical treatment may result in better outcomes in terms of pain relief, vertebral function, and complications. However, the surgical timing still needs to be considered in light of the specific circumstances of each patient, as well as various other factors.

## Data Availability

The raw data supporting the conclusions of this article will be made available by the authors, without undue reservation.

## References

[1] Johnell O Kanis JA. An estimate of the worldwide prevalence and disability associated with osteoporotic fractures. Osteoporos Int. (2006) 17(12):1726–33. 10.1007/s00198-006-0172-4 16983459

[2] Longo G Loppini M Denaro L Maffulli N Denaro V. Conservative management of patients with an osteoporotic vertebral fracture: a review of the literature. J Bone Joint Surg Br. (2012) 94(2):152–7. 10.1302/0301-620X.94B2.26894 22323677

[3] Wang H Sribastav SS Ye F Yang C Wang J Liu H Comparison of percutaneous vertebroplasty and balloon kyphoplasty for the treatment of single level vertebral compression fractures: a meta-analysis of the literature. Pain Physician. (2015) 18(3):209–22. 26000665

[4] Long Y Yi W Yang D. Advances in vertebral augmentation systems for osteoporotic vertebral compression fractures. Pain Res Manag. (2020) 2020:3947368. 10.1155/2020/3947368 33376566 PMC7738798

[5] Kamei S Noguchi T Shida Y Okafuji T Yokoyama K Uchiyama F The safety and efficacy of percutaneous vertebroplasty for patients over 90 years old. Jpn J Radiol. (2019) 37(2):178–85. 10.1007/s11604-018-0797-1 30506449

[6] Pan M Ge J Li Q Li S Mao H Meng B Percutaneous vertebral augmentation in special genant IV osteoporotic vertebral compression fractures. J Orthop Translat. (2020) 20:94–9. 10.1016/j.jot.2019.07.002 31908939 PMC6938938

[7] Lou S Shi X Zhang X Lyu H Li Z Wang Y. Percutaneous vertebroplasty versus non-operative treatment for osteoporotic vertebral compression fractures: a meta-analysis of randomized controlled trials. Osteoporos Int. (2019) 30(12):2369–80. 10.1007/s00198-019-05101-8 31375875

[8] Kobayashi K Shimoyama K Nakamura K. Percutaneous vertebroplasty immediately relieves pain of osteoporotic vertebral compression fractures and prevents prolonged immobilization of patients. Eur Radiol. (2005) 15(2):360–7. 10.1007/s00330-004-2549-0 15662480

[9] Mathis JM Barr JD Belkoff SM Barr MS Jensen ME Deramond H. Percutaneous vertebroplasty: a developing standard of care for vertebral compression fractures. Am J Neuroradiol. (2001) 22(2):373–81. 11156786 PMC7973930

[10] Zhou X Meng X Zhu H Zhu Y Yuan W. Early versus late percutaneous kyphoplasty for treating osteoporotic vertebral compression fracture: a retrospective study. Clin Neurol Neurosurg. (2019) 180:101–5. 10.1016/j.clineuro.2019.03.029 30953973

[11] He B Zhao J Zhang M Jiang G Tang K Quan Z. Effect of surgical timing on the refracture rate after percutaneous vertebroplasty: a retrospective analysis of at least 4-year follow-up. BioMed Res Int. (2021) 2021:1–7. 10.1155/2021/5503022 34873571 PMC8643249

[12] Guan H Yang H Mei X Liu T Guo J. Early or delayed operation, which is more optimal for kyphoplasty? A retrospective study on cement leakage during kyphoplasty. Injury. (2012) 43(10):1698–703. 10.1016/j.injury.2012.06.008 22769978

[13] Liu J Li X Tang D Li X Yao M Yu P Comparing pain reduction following vertebroplasty and conservative treatment for osteoporotic vertebral compression fractures: a meta-analysis of randomized controlled trials. Pain Phys. (2013) 16(5):455–64. 24077192

[14] Lin F Zhang Y Song X Niu Y Su P Hua J Percutaneous kyphoplasty to relieve the rib region pain in osteoporotic thoracic vertebral fracture patients without local pain of fractured vertebra. Pain Physician. (2023) 26(1):53–9. 36791294

[15] Lee HM Park SY Lee SH Suh SW Hong JY. Comparative analysis of clinical outcomes in patients with osteoporotic vertebral compression fractures (OVCFs): conservative treatment versus balloon kyphoplasty. Spine J. (2012) 12(11):998–1005. 10.1016/j.spinee.2012.08.024 23026068

[16] Yu D Liu Z Wang H Yao R Li F Yang Y Treatment of elderly patients with acute symptomatic OVCF: a study of comparison of conservative treatment and percutaneous kyphoplasty. Front Surg. (2022) 9. 10.3389/fsurg.2022.942195 PMC9326083 35910474

[17] Ehsanian R To J Koshkin E Petersen TR Berti A Rivers WE A single-center retrospective analysis investigating the effect of timing of vertebral augmentation on pain outcomes. Pain Physician. (2022) 25(9):E1423–31. 36608014

[18] Liu T Li Z Su Q Hai Y. Cement leakage in osteoporotic vertebral compression fractures with cortical defect using high-viscosity bone cement during unilateral percutaneous kyphoplasty surgery. Medicine (Baltimore). (2017) 96(25):e7216. 10.1097/MD.0000000000007216 28640112 PMC5484220

[19] Tang B Cui L Chen X Liu Y. Risk factors for cement leakage in percutaneous vertebroplasty for osteoporotic vertebral compression fractures: an analysis of 1456 vertebrae augmented by low-viscosity bone cement. Spine (Phila Pa 1976). (2021) 46(4):216–22. 10.1097/BRS.0000000000003773 33156285

[20] Wang L Zhang C Liang H Huang T Zhong W Zhao Z. Cement leakage in percutaneous vertebroplasty for spinal metastases: a retrospective study of risk factors and clinical outcomes. World J Surg Oncol. (2022) 20(1):112. 10.1186/s12957-022-02583-5 35387653 PMC8988338

[21] Hou JG Zhang N Chen GD. Factors affecting cement leakage in percutaneous vertebroplasty: a retrospective cohort study of 309 patients. Eur Rev Med Pharmacol Sci. (2023) 27(9):3877–86. 10.26355/eurrev_202305_32293 37203859

[22] Zhu S Zhong Z Wu Q Chen JT. Risk factors for bone cement leakage in percutaneous vertebroplasty: a retrospective study of four hundred and eighty five patients. Int Orthop. (2016) 40(6):1205–10. 10.1007/s00264-015-3102-2 26753843

[23] Zhang J He X Fan Y Du J Hao D. Risk factors for conservative treatment failure in acute osteoporotic vertebral compression fractures (OVCFs). Arch Osteoporos. (2019) 14(1):24. 10.1007/s11657-019-0563-8 30806831

